# Salami Publication in Qualitative Research: An Ethical Challenge

**Published:** 2019-08

**Authors:** Roksana JANGHORBAN, Fatemeh AZARKISH

**Affiliations:** 1. Community-Based Psychiatric Care Research Center, Department of Midwifery, School of Nursing and Midwifery, Shiraz University of Medical Sciences, Shiraz, Iran; 2. Department of Midwifery, School of Nursing and Midwifery, Iranshahr University of Medical Sciences, Iranshahr, Iran

## Dear Editor-in-Chief

Ethics is commonly defined as “norms of conduct” that is used as a benchmark for measuring acceptability of behavior. Ethical conduct has become one of interested subjects in all arenas such as research. Adherence to ethical norms in research could promote research accuracy, essential values in collaborative work, “public support for research” and researchers’ accountability to the public. Ethical misconduct means that highly unethical or illegal behavior in research done intentional, knowing or reckless (1). In comparison of biomedical research, the application of some research ethics to examine and evaluate qualitative research has been less considered (2). One of the issues is salami publication.

Salami publication means that extracts several articles from one data set for increasing publication counts (3). It is considered as ethical misconduct by the Committee on Publication Ethics (COPE), but it could be allowed in large epidemiological studies and randomized control trials with large amount of data, and when manuscripts derived from a study published in different journals for different audiences. However, in the last case, authors have responsibility of editors’ awareness in terms of necessary information for evaluation of subject accuracy and decision to publish (4).

Salami slicing originates from academic pressure to publish, authors’ intention to “artificially enlarge” number of their publication, “carrier advancement”, and increase probability of citations (3, 4). Consequences of the unethical phenomena could be distortion of the literature, skewed “scientific database”, waste of editors’, reviewers’, and readers’ time. Salami publication has serious outcome in quantitative studies especially clinical trials due to distort pool result of them in systematic reviews and meta-analysis and its application in guidelines production and clinical practice (4).

Qualitative research has different nature from quantitative studies including role of researcher as key instrument for data collection and investigation of meaning of very personal experiences. Most ethical considerations arise from the specific characteristics of qualitative research. They consist of prevention of participant vulnerability, respect to privacy, maintaining confidentiality and anonymity, obtaining informed consent, and having ethical and interactive relationship between researchers and participants (2, 5). Most of ethical considerations focus on challenges from designing to implementation, not in dissemination of findings as articles (5). Therefore critique of salami publication in qualitative studies is limited.

Detection of salami publication is so complex because there are no objective ways and software applications for checking (4). The ethical misconduct could be more detectable in clinical trials articles than qualitative studies due to mandatory registration of trials in a clinical trial registry. Editors can check trial records of submitted manuscripts for detecting salami publication by a unique identification code given to each clinical study (6). Additionally, temptation of salami publication in qualitative research could be increased especially in manuscripts are extracted from Ph.D. students’ thesis to enlarge publication numbers and citations (3). For instance, it may lead to produce multiple qualitative manuscripts written by content analysis approach from a study with grounded theory methodology. Impact of the act could be so harmful because aim of qualitative research is provision of thick description of participants’ experiences and phenomenon under exploration (7). On the other hand, another idea focuses on ethical problems of dissemination of qualitative findings from one data set in one article due to provide a superficial description of participants’ perceptions owing to word restrictions for articles in journals (8).

Salami slicing could be a serious and less detectable threat to publication ethics in qualitative research. Authors could avoid it by having commitment to research protocol from designing to reporting findings, considering to publication aim, intended audience, and word limit of journals, and providing full disclosure of their related works. Additionally, the idea to institutionalize culture of research ethics, decrease the pressure to publish in academic institutions and register of research protocol in a qualitative registry may help increase integrity, openness, and honesty of work and reduce the incidence of ethical misconducts such as salami publication.
